# Long-term survival in low-grade endometrial stromal sarcoma with childbirth and multidisciplinary treatment: a case report

**DOI:** 10.1186/s13256-015-0719-0

**Published:** 2015-10-20

**Authors:** Osamu Maeda, Suzuko Moritani, Shu Ichihara, Takami Inoue, Yutaka Ishihara, Shinichi Yamamoto, Makoto Ito, Yoko Matsumura, Kumiko Sugiyama, Maiko Horio, Ikuyo Kondo

**Affiliations:** 1 Department of Obstetrics and Gynecology, Meijo Hospital, Sannomaru 1-3-1, Naka-ku, Nagoya, 460-0001 Japan; 2 Department of Pathology, National Hospital Organization, Nagoya Medical Center, Nagoya, Japan; 3 Kishokai Medical Corporation, Inazawa, Aichi Japan; 4 Department of Gynecology, Inazawa Municipal Hospital, Inazawa, Aichi Japan; 5 Department of Obstetrics and Gynecology, Kariya-Toyota General Hospital, Kariya, Aichi Japan; 6 Department of Pathology and Laboratory Medicine, Kariya-Toyota General Hospital, Kariya, Aichi Japan

**Keywords:** Chemotherapy, Childbirth, Hormone therapy, Low-grade endometrial stromal sarcoma, Surgery

## Abstract

**Introduction:**

Low-grade endometrial stromal sarcoma is very rare and difficult to diagnose in the early stage. A standard treatment has not been established. In this case report of a patient with long-term survival, we describe an effective treatment for advanced low-grade endometrial stromal sarcoma.

**Case presentation:**

A 24-year-old Japanese woman who presented with prolonged menstruation was diagnosed with leiomyoma on the basis of a specimen resected transvaginally. She underwent ten resections in 10 years without a malignancy diagnosis. During this period, she gave birth. At age 34 years, she visited our hospital, complaining of lower abdominal pain. A 10cm tumor was detected behind her uterus. The disease was diagnosed as an advanced malignant ovarian tumor before surgery. A laparotomy was performed, with many remnants left in the abdominal cavity. The final diagnosis was advanced low-grade endometrial stromal sarcoma. After 12 cycles of gemcitabine and docetaxel combination chemotherapy, the tumor disappeared completely. A retrospective pathological review of the specimens resected transvaginally showed that the tumors included low-grade endometrial stromal sarcoma elements. When the patient was age 42 years, the sarcoma recurred. It was detected around the right diaphragm and liver. Despite administration of gemcitabine and docetaxel, ascites and pleural effusion accumulated. Administration of medroxyprogesterone acetate, leuprorelin acetate, and anastrozole gradually reduced the ascites and pleural effusion. In addition to the three hormone drugs, 18 cycles of paclitaxel and carboplatin were administered. The patient recovered from her critically ill state and is currently alive with reduced tumor at age 45 years.

**Conclusions:**

Our patient with low-grade endometrial stromal sarcoma whose disease began in her youth gave birth and experienced long-term survival with surgery, chemotherapy, and hormone therapy.

## Introduction

Low-grade endometrial stromal sarcoma (LGESS) is very rare and tends to grow slowly. It develops in the supporting connective tissue (stroma) [1, 2]. Koss and colleagues stated that endometrial stromal sarcoma constitutes only 0.2% of all uterine cancers and described long-term survival of ten patients with this disease. The prognosis of LGESS is better than that of other uterine sarcomas [3]; however, LGESS is difficult to diagnose in the early stage or before surgery. A standard treatment is not established, so women with advanced-stage LGESS may undergo surgery, hormone treatment, chemotherapy, or radiation as salvage therapy.

We report a case of a patient with LGESS who has experienced long-term survival. She gave birth in the early stage of LGESS, received chemotherapy after surgery in the advanced stage, and was revived by hormone therapy and additional chemotherapy in the recurrent stage. We hope this case report plays a role in establishing effective treatment for LGESS.

## Case presentation

### Transvaginal tumor resections as benign tumors and childbirth in the early stage

We report a case of a 24-year-old Japanese woman, gravida 0, who had no relevant past medical history or family history when she became ill. She complained of prolonged menstruation and was examined with Cusco’s speculum. The patient presented with a uterine cervical tumor and was diagnosed with uterine leiomyoma. She underwent her first transvaginal resection of the tumor with a myomectomy and endometrial curettage. The pathological diagnosis was leiomyoma and endometrium, no malignancy, as a benign tumor. She underwent four additional tumor resections: polypectomy, two myoma resections, and a submucosal leiomyoma resection via hysteroscopy. The resections were performed at intervals of 10 months, 9 months, 9 months, and 6 months, respectively. After these resections, she gave birth to a 3396g boy via cesarean section at age 28 years. During her pregnancy, she was diagnosed with oligoamnios. The birth took place at the 39th week of pregnancy. At age 30 years, the patient developed a sixth tumor (a fibroid in statu nascendi), which was removed by exeresis at age 30 years. Four subsequent tumor resections were performed, at intervals of 23 months, 4 months, 12 months, and 3 months, respectively. When the seventh tumor was resected by polypectomy at age 32 years, the pathology report revealed for the first time the high density of the stroma and the necessity of careful follow-up. At the time these ten tumor resections were performed (five before and five after childbirth), the tumors were not diagnosed as malignant. The correct diagnosis was obtained by analysis of advanced-stage surgical specimens. The discovery of minimal elements of LGESS by a retrospective study of the transvaginally resected tumors led to a correct diagnosis of early-stage LGESS.

### Treatment in the advanced stage beginning with abdominal pain

**Fig. 1:**
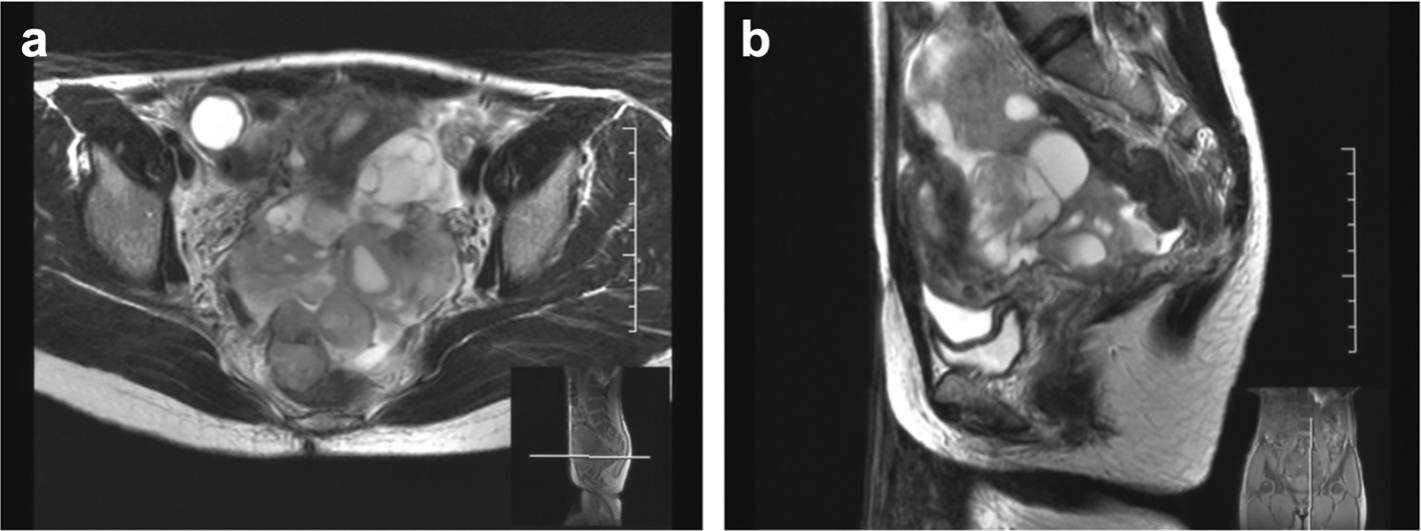
Pelvic magnetic resonance imaging scans showing advanced low-grade endometrial stromal sarcoma 10cm in diameter behind the uterus with signal intensities from low to high. **a** T2-weighted axial slice. **b** T2-weighted sagittal slice

The patient visited our hospital, complaining of lower abdominal pain at age 34 years and was found to have a tumor 10cm in diameter behind the uterus (Fig. 1), which was diagnosed as an advanced malignant ovarian tumor before surgery. Total abdominal hysterectomy, bilateral salpingo-oophorectomy, and resection of peritoneal dissemination were performed. The tumor was classified as International Federation of Gynecology and Obstetrics 1982 stage VIb for uterine corpus cancer and Union for International Cancer Control 1990 tumor, node, metastasis stage pT4NxM1 on the basis of the finding of tumor spreading in the peritoneal cavity. The histological result revealed LGESS of the uterine corpus. A great amount of bulky, disseminated tumor lingered in the abdominal cavity, particularly on the serosa of the rectum and descending colon. After the operation, gemcitabine (800mg/body surface area, Day 1 and 8) and docetaxel (60mg/body surface area, Day 8) combination chemotherapy (GD) was started with informed consent. A subcommittee of our institutional review board reviewed the case and gave approval to treat this patient with GD chemotherapy. Six cycles of GD were administered every 3 weeks, followed by six cycles every 3 months (age 36 years). After nine cycles of chemotherapy, the patient developed ileus and underwent surgery. She had a complete response to chemotherapy, and no visible tumor remained in the abdominal cavity. No recurrent tumor was detected for the next 4 years, and the patient did not undergo any treatment during that time.

When the patient reached the age of 40 years, a new tumor was detected in the pelvic cavity. She underwent surgery to remove the recurrent tumor. The tumor, 2.5cm in diameter disseminated on the ileum, was resected. Numerous small disseminations remained. Five cycles of GD every 3 weeks were administered after surgery. The sixth cycle of GD was discontinued because the patient’s neutropenia worsened to a grade 4 adverse event as defined by Common Terminology Criteria for Adverse Events (CTCAE) version 3, even after granulocyte colony-stimulating factor was administered. No recurrent tumor was detected for the next 18 months.

### Revival from recurrence between right diaphragm and liver surface

**Fig. 2:**
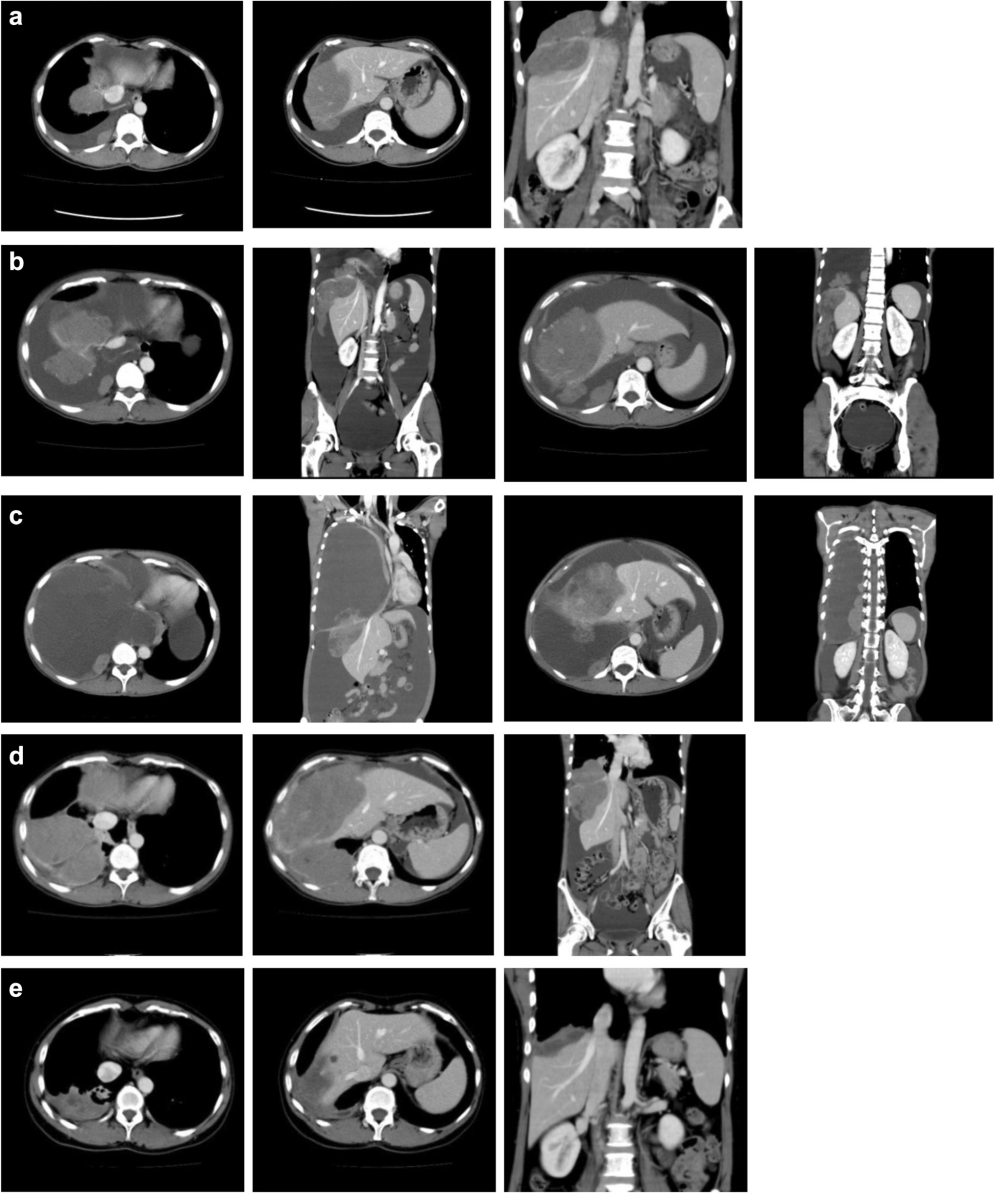
Enhanced computed tomographic scans obtained after tumor recurrence around the right diaphragm and liver surface. **a** Recurrent tumor found before administering gemcitabine and docetaxel combination chemotherapy. **b** Bleeding in the abdominal cavity after administering gemcitabine and docetaxel combination chemotherapy. **c** Four weeks after starting medroxyprogesterone acetate and one week after starting leuprorelin acetate. **d** Before administering paclitaxel and carboplatin combination chemotherapy and after administering medroxyprogesterone acetate, leuprorelin acetate, and anastrozole. **e** After administering 18 cycles of paclitaxel and carboplatin combination chemotherapy and medroxyprogesterone acetate, leuprorelin acetate, and anastrozole

A small accumulation of fluid was detected in the pouch of Douglas when the patient was age 42 years. Computed tomography (CT) revealed recurrence of the tumor in the form of many disseminations in the right pleura, peritoneum, right diaphragm pleural and peritoneal surface, and liver surface (Fig. 2a). Although three cycles of GD at the initial dose were administered, the disease progressed and shock was induced by abdominal cavity bleeding (Fig. 2b). Infusion and blood transfusion were started with hospitalization to treat bleeding shock. Medroxyprogesterone acetate (MPA) was administered at a daily dose of 600mg. Although the bleeding in the patient’s abdominal cavity stopped, her pleural effusion and ascites did not decrease. Because the effect of MPA was not enough, a gonadotropin-releasing hormone analogue (leuprorelin acetate) at a dose of 3.75mg/body surface area once every 28 days was administered in addition to MPA. The increase in both the pleural effusion and ascites accelerated, and thoracic bleeding induced shock and dyspnea on the 18th day after leuprorelin acetate was first administered (Fig. 2c). The pleural effusion began to decrease at 1 month after leuprorelin acetate was first administered. Because the patient’s ascites were not decreasing, an aromatase inhibitor (anastrozole) 1mg/day was administered in addition to MPA and leuprorelin acetate. After beginning treatment with anastrozole, the patient’s ascites decreased. She was discharged from the hospital after 116 days.

Three months later, the tumor had enlarged between the liver and diaphragm and had spread to the right pleura (Fig. 2d). Paclitaxel at a dose of 170mg/m^2^ and carboplatin (area under the curve =4) combination chemotherapy (TC) was started with MPA, leuprorelin acetate, and anastrozole. The dose of TC was reduced because during the time five cycles of GD were administered after the recurrent tumor on the ileum was resected the patient’s neutropenia gradually worsened to CTCAE grade 4. The tumor size and state were confirmed by CT after every three cycles of chemotherapy. The tumor gradually reduced, and the liver returned to the normal position. Chemotherapy was stopped after 18 cycles (Fig. 2e). The patient is currently alive and being treated with MPA, leuprorelin acetate, and anastrozole (age 45 years at the time of this report).

### Pathological review

**Fig. 3:**
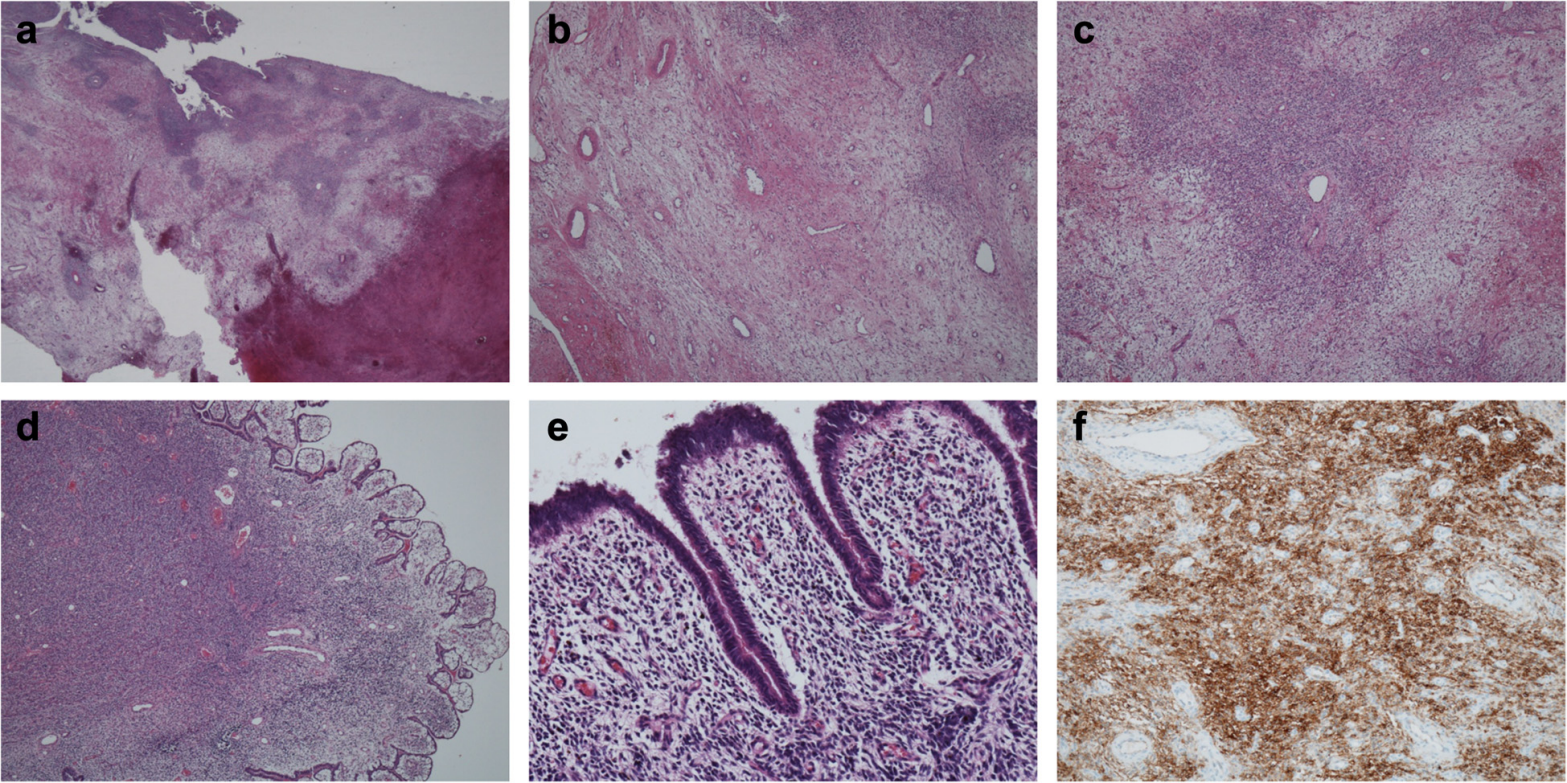
Histological findings of transvaginal resection. **a** Polypoid lesion with surface erosion and hemorrhage (*lower right*). **b** The stroma was loosely fibrous with areas of edema. Scattered capillaries and small vessels were seen. **c** Focal area of hypercellularity. Short spindle stromal cells surround a small blood vessel. **d** The seventh transvaginal resection. The stroma is markedly hypercellular. **e** High-power magnification of image shown in (**d**). **f** Cluster of differentiation 10 immunostaining of the first transvaginal resection

The first, sixth, and ninth tumors transvaginally resected in the early stage showed similar histopathological findings. The tumors had a polypoid appearance with various degrees of surface erosion (Fig. 3a). The surfaces were covered by non-atypical columnar epithelium of endocervical or endometrial type. The stroma was edematous, loosely fibrous, and paucicellular, often containing capillaries and small vessels recapitulating inflammatory granulation tissue (Fig. 3b). There were a few foci of mild stromal hypercellularity, where small, short spindle stromal cells surrounded the blood vessels (Fig. 3c). The seventh, eighth, and tenth transvaginally resected tumors were clinically diagnosed as endocervical or endometrial polyps, and they had wide areas of stromal hypercellularity, which is unusual for conventional endocervical or endometrial polyps (Fig. 3d, e). Although several suspicious findings were present, it was difficult to diagnose LGESS on the basis of only transvaginal resection of the tumors.

The surgically resected uterus in the advanced stage had multiple polypoid excrescences in the lower uterine segment. Examination of cut sections revealed multiple nodular lesions in the muscular layer of the uterine corpus and paraovarian connective tissue. The cut surfaces of these tumors were whitish yellow and had a monotonous solid appearance. Multiple peritoneal tumors ranging from 20mm to 60mm in diameter had similar macroscopic findings.

**Fig. 4:**
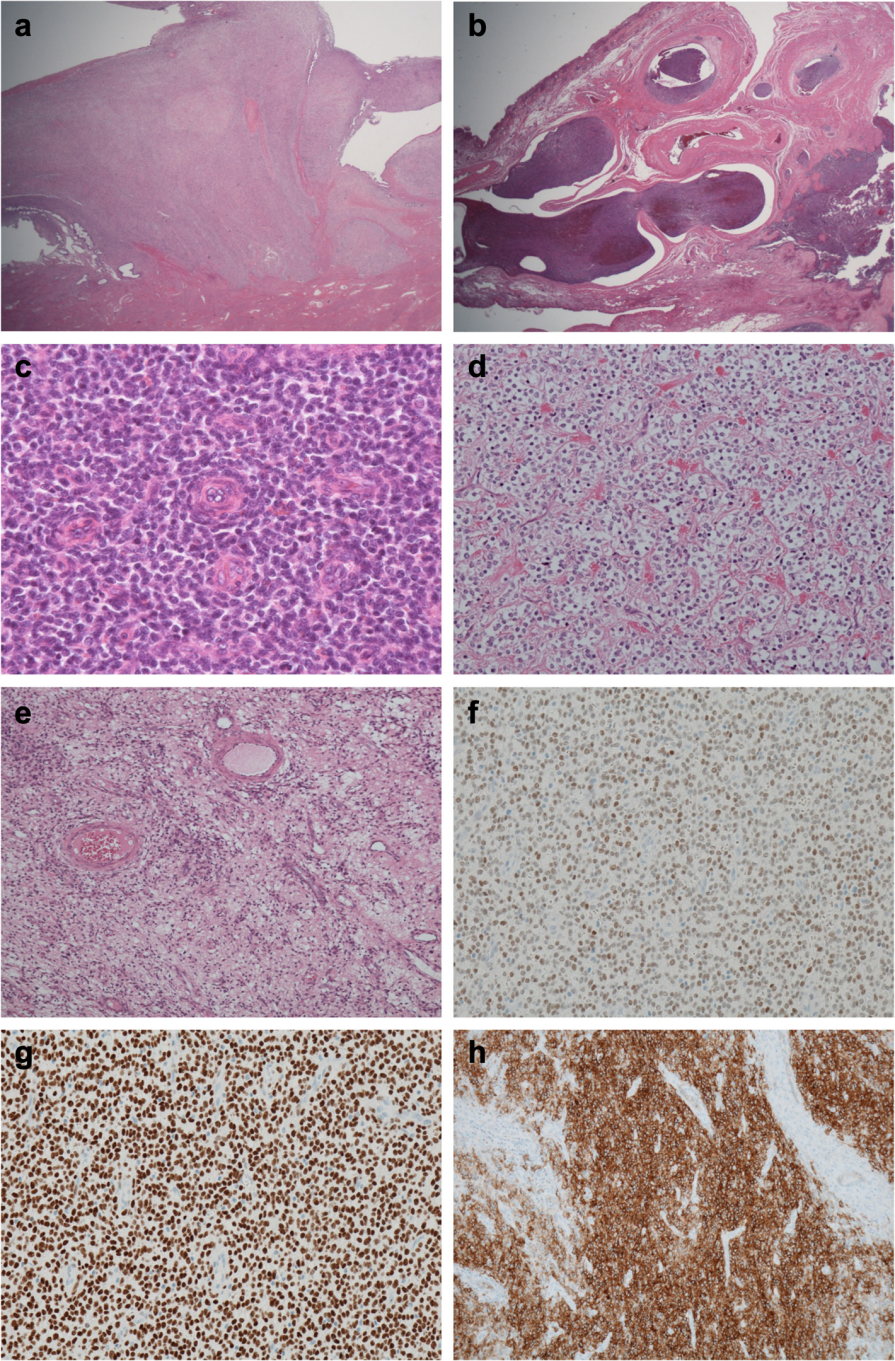
Histological findings of surgically resected specimens. **a** Endometrial lesion of the lower uterine segment. The tumor grew exophytically and invaded the muscular layer with tongue-like projections. **b** Prominent intravascular extension in the paraovarian connective tissue. **c** Oval tumor cells surround arterioles in a whorl-like pattern. **d** In some areas, tumor cells have abundant clear cytoplasm and show an epithelioid appearance. **e** Foci of hypocellular fibrous areas show similarity to the lesions of the pre-operative transvaginal resections. **f**, **g**, and **h** Immunohistochemical staining of the surgically resected tumor. **f** Estrogen receptor. The tumor cells are diffusely positive with weak to moderate signal intensity. **g** Progesterone receptor. The tumor cells are diffusely and strongly positive. **h** Cluster of differentiation 10. The tumor cells are diffusely positive

Histologically, the polypoid endometrial lesions invaded the muscle layer with pushing margins and multiple tongue-like projections (Fig. 4a). Prominent intralymphatic tumor extensions were noted, especially in the paraovarian connective tissue (Fig. 4b). The tumor consisted of a monotonous proliferation of oval to short spindle cells with indistinct cytoplasm (Fig. 4c). The spindle cells often surrounded small arterioles in a concentric pattern. Some cells had abundant clear cytoplasm (Fig. 4d). In some areas, the stroma was edematous and cellularity was low (Fig. 4e). Such areas were similar to the pathological findings of the first, sixth, and ninth transvaginal polypectomy specimens.

Immunohistochemically, the tumor cells of the surgically resected specimens were diffusely positive for estrogen receptor, progesterone receptor, and cluster of differentiation 10 (CD10) (Fig. 4f, g, and h). The staining intensity of progesterone receptor and CD10 was strong, and that of estrogen receptor was weak to moderate. CD10 was also positive in the spindle cell of the pre-operative specimens of the transvaginal resections (Fig. 3f). The series of lesions, including those from the ten transvaginal resections and hysterectomy, were finally diagnosed as LGESS. Focal stromal hypercellularity surrounding small blood vessels seen in the first ten resected tumors was retrospectively considered the characteristic finding of LGESS. The recurrent intestinal serosal, peritoneal, and hepatic lesions showed essentially the same histological findings.

## Discussion

LGESS is a rare disease. Several case reports describing pregnancy after contracting this disease have been published [4–6]. In our present report, we describe a case of childbirth amid repeated tumor resections. Furthermore, our patient was treated after her condition worsened to the advanced stage. She underwent surgery, hormone therapy, and combination chemotherapy. All of these treatment modalities led to a good response and long-term survival.

The tumors, which had the appearance of uterine myomas or cervical polyps, grew repeatedly during the early stage over a period of 10 years. The pathological diagnosis of LGESS could not be made until a hysterectomy was performed. The usual pathological features of LGESS are tongue-like myometrial invasions with prominent lymphatic involvement and a whorled pattern of oval to short spindle tumor cells around the arterioles. When these characteristic features are identified, the pathological diagnosis is usually straightforward. However, myometrial invasion is difficult or almost impossible to identify in myomectomy or polypectomy specimens. In addition to the whorled pattern, LGESS has a wide spectrum of less common histological patterns, including fibroblastic or myofibroblastic appearance, epithelioid arrangement of the tumor cells with abundant clear or eosinophilic cytoplasm, hypocellular myxoid or fibrous change, sex cord–like elements, glandular differentiation, and pseudopapillary pattern [7]. In the specimens of the transvaginal resections of our patient, significant areas of the lesions showed a hypocellular granulation tissue-like or fibrous appearance with less prominent whorled patterns characteristic of LGESS. Those findings are the reason for the difficulty in pre-operative pathological diagnosis of LGESS in our patient. Fortunately, the delay of the pathological diagnosis, with avoidance of hysterectomy, allowed the patient to give birth.

It was difficult to find effective treatments in a search of the literature because articles on this topic have been published only sporadically and report cases only at the time the tumor had worsened to the advanced stage. Although surgery, radiation therapy, chemotherapy, and progestin have been reported as treatments for LGESS, the effect of these treatments has been limited and ineffective in advanced cases. When the next treatment was investegated after the first abdominal surgery, GD had been reported as an effective therapy for unresectable leiomyosarcoma in a phase II trial [8]. GD was considered the treatment of choice in our patient, although the detailed histological type in her pathology reports was different from leiomyosarcoma. Possible use of ifosfamide, doxorubicin, and cisplatin combination therapy (IAP) was also discussed. There was only one case report of high-grade endometrial stromal sarcoma [9] that described this therapy, which would have produced more severe side effects than GD. Because GD could be administered in the outpatient clinic, it was considered that GD would be better than IAP for our patient from the standpoint of enabling her to have a good quality of life. We also discussed possible use of MPA hormone therapy. Although a few single-case reports about leuprolin acetate [10, 11] or letrozole [12] had been reported before our patient’s case, satisfactory descriptions about treatment with these drugs accumulated later [13, 14]. Because of the severity of our patient’s disease and the many tumor remnants in her abdominal cavity, GD was selected rather than MPA as the treatment of choice. Administration of GD was greatly effective and led to two complete responses. Until a sarcoma recurred around her liver and right diaphragm, our patient survived tumor-free for approximately 8 years after she first reported abdominal pain and was treated with surgery followed by GD chemotherapy. During this 8-year period, progress was made in treatment options for LGESS, and leuprorelin acetate and anastrozole were reported as effective drugs.

In the second recurrence of our patient’s disease, GD treatment did not produce a response. It was considered that the tumor had acquired anti-cancer drug resistance against GD because of repeated GD administration. Because MPA as a next treatment was not effective enough, leuprorelin acetate was administered in addition to MPA. After administration of leuprorelin acetate, the patient’s pleural effusion and bleeding accelerated. Leuprorelin acetate caused a flare-up of gonadotropin secretion. However, the bleeding stopped after the period of flare-up of gonadotropin secretion, and the pleural effusion decreased gradually. There was no change in the patient’s ascites, though, so anastrozole was administered in addition to MPA and leuprorelin acetate. This treatment was effective in reducing the ascites, and the patient could be discharged from the hospital. The three drugs did not produce side effects, except for bleeding caused by leuprorelin acetate.

In the next step, TC was administrated in addition to hormone therapy. TC was selected because it is a standard treatment for uterine corpus cancer and is easy to administer. TC with MPA, leuprorelin acetate, and anastrozole is effective for patients with LGESS resistant to GD chemotherapy. Although our patient experienced the typical side effects of chemotherapy, such as alopecia, bone marrow suppression, and numbness, she underwent 18 cycles of TC chemotherapy without any more severe side effects than were expected. A reduced dose would be good for the patient to continue the repeated chemotherapy. The patient has survived more than 10 years after the disease spread throughout her abdominal cavity. However, this chemotherapy and hormone therapy will cease to be effective when the patient eventually develops resistance to the anti-cancer drugs currently used to treat her. A new treatment option is desired for the next recurrence of LGESS.

## Conclusions

The diagnosis of LGESS in the early stage is very difficult only on the basis of transvaginally resected specimens. Our patient gave birth in the early stage of the disease. Long-term survival was accomplished via surgery, GD in the advanced stage, and TC combined with hormone therapy for the GD-resistant tumor in the recurrent stage. This report should give hope to patients with LGESS. We hope that combination chemotherapy together with hormone therapy can be established as an effective treatment for advanced LGESS.

## Consent

Written informed consent was obtained from the patient for publication of this case report and accompanying images. A copy of the written consent is available for review by the Editor-in-Chief of this journal.

## Abbreviations

CD10: Cluster of differentiation 10
CT: Computed tomography
CTCAE: Common Terminology Criteria for Adverse Events
GD: Gemcitabine and docetaxel combination chemotherapy
IAP: Ifosfamide, doxorubicin, and cisplatin combination therapy
LGESS: Low-grade endometrial stromal sarcoma
MPA: Medroxyprogesterone acetate
TC: Paclitaxel and carboplatin combination chemotherapy
